# 43. SARS-CoV-2 Cycle threshold (Ct) values predict future COVID-19 cases

**DOI:** 10.1093/ofid/ofab466.043

**Published:** 2021-12-04

**Authors:** Matthew Phillips, David Quintero, Susan Butler-Wu

**Affiliations:** 1 Los Angeles County + University of Southern California Medical Center, Los Angeles, California; 2 Keck School of Medicine of USC, CA

## Abstract

**Background:**

The threat of surging COVID-19 cases prompted many hospitals in the United States to preemptively suspend elective procedures throughout the pandemic. Utilizing samples from a large hospital in Los Angeles, we sought to determine if temporal trends in SARS-CoV-2 Cycle threshold (Ct) values (proxy for viral RNA loads) were predictive for the number of future COVID-19 cases.

**Methods:**

Nasopharyngeal specimens on symptomatic patients and asymptomatic admissions were tested using the Xpert Xpress SARS-CoV-2 and SARS-CoV-2/Flu/RSV assays (Cepheid). Ct values for all SARS-CoV-2 detections between October 2020 to March 2021 were compiled for analysis.

**Results:**

A total of 2,114 SARS-CoV-2-positive samples were included. The number of tests performed per week increased dramatically in December peaking the first week of January before returning to pre-surge numbers by Mid-February. Ct values fell during this same period with values in December and January (25.6±7.8 and 27±7.9, respectively) significantly lower than those of the other months (30±9.3 to 37.7±6.3). Average weekly Ct values for all patients were significantly, negatively correlated with the number of tests run the following week (R= -0.71, P< 0.001) and two weeks later (R= -0.75, P< 0.0001). Ct values for patients who were asymptomatic at the time of testing most strongly correlated with total number of tests performed one month later (R= -0.86, P< 0.0001).

Average weekly Ct values and number of test run

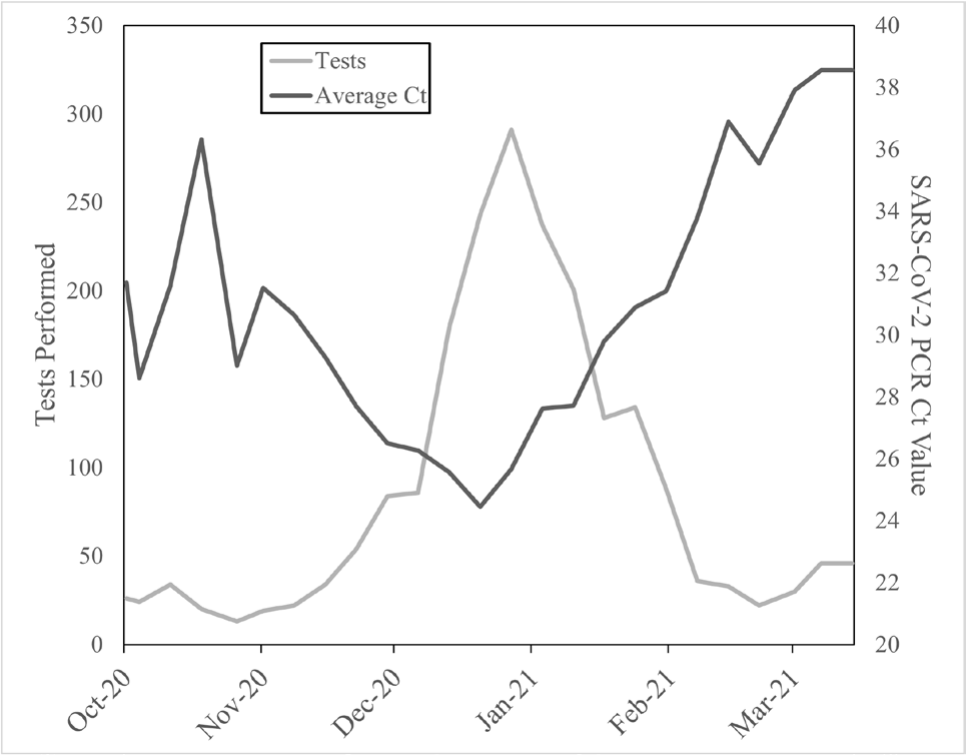

As cases (light grey) increased during December and January, there was a significant decrease in Ct values (dark grey) during that same time period.

Average Ct values are a leading indicator of cases

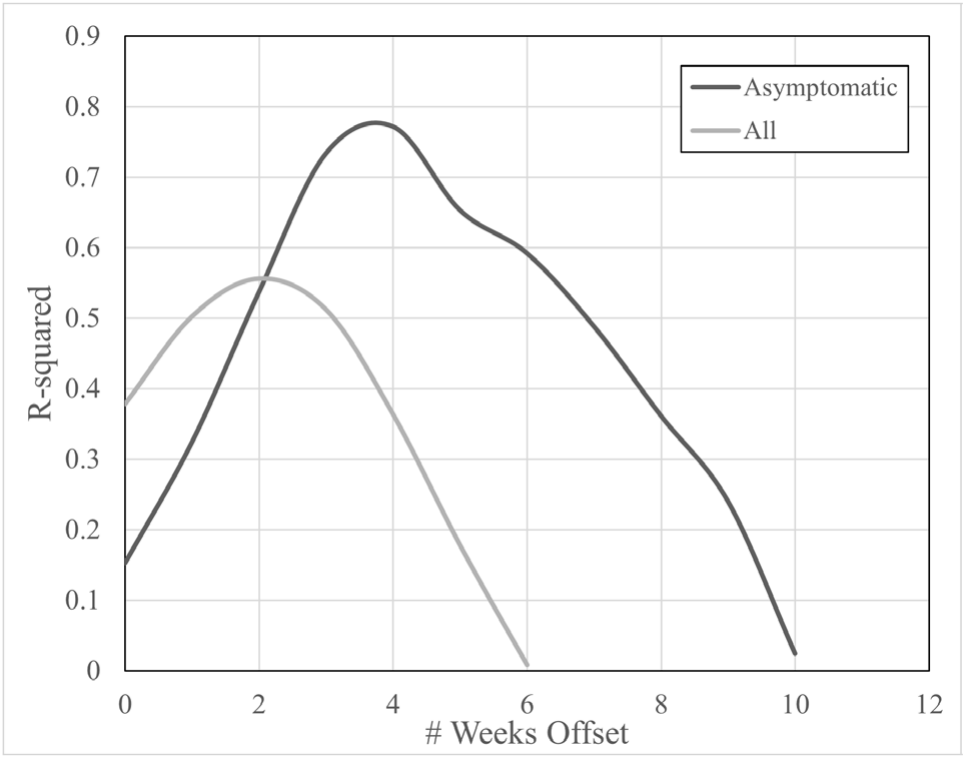

Average weekly Ct values for all patients (light grey) were significantly, negatively correlated with the number of tests run the following week (R= -0.71, P<0.001) and two weeks later (R= -0.75, P<0.0001). Ct values for patients who were asymptomatic at the time of testing (dark grey) most strongly correlated with total number of tests performed one month later (R= -0.86, P<0.0001).

**Conclusion:**

Lower Ct values, representing higher levels of viral RNA, have been associated with risk of intubation and infectivity. During the winter surge, we observed significantly lower Ct values suggesting that the increased transmission and morbidity of COVID-19 was temporarily associated with higher viral loads. Interestingly, Ct values for asymptomatic patients were most strongly associated with number of cases observed 1 months in the future, suggesting that asymptomatic viral load may be a leading indicator for forthcoming outbreaks. Given this association, Ct values may be a useful tool for predicting regional outbreaks of COVID-19 and more judicious cessation of elective procedures.

**Disclosures:**

**All Authors**: No reported disclosures

